# Vibrational Spectroscopy for In Vitro Monitoring Stem Cell Differentiation

**DOI:** 10.3390/molecules25235554

**Published:** 2020-11-26

**Authors:** Francesca Ravera, Esen Efeoglu, Hugh J. Byrne

**Affiliations:** 1 School of Physics and Clinical and Optometric Sciences, Technological University Dublin, City Campus, 8 Dublin, Ireland; 2 FOCAS Research Institute, Technological University Dublin, City Campus, 8 Dublin, Ireland; hugh.byrne@tudublin.ie; 3 School of Biomolecular and Biomedical Science, University College Dublin, Belfield, 4 Dublin, Ireland; esenefeoglu@gmail.com

**Keywords:** stem cell technology, vibrational spectroscopy, Raman spectroscopy, FTIR absorption spectroscopy, stem cell differentiation, spectral markers

## Abstract

Stem cell technology has attracted considerable attention over recent decades due to its enormous potential in regenerative medicine and disease therapeutics. Studying the underlying mechanisms of stem cell differentiation and tissue generation is critical, and robust methodologies and different technologies are required. Towards establishing improved understanding and optimised triggering and control of differentiation processes, analytical techniques such as flow cytometry, immunohistochemistry, reverse transcription polymerase chain reaction, RNA in situ hybridisation analysis, and fluorescence-activated cell sorting have contributed much. However, progress in the field remains limited because such techniques provide only limited information, as they are only able to address specific, selected aspects of the process, and/or cannot visualise the process at the subcellular level. Additionally, many current analytical techniques involve the disruption of the investigation process (tissue sectioning, immunostaining) and cannot monitor the cellular differentiation process in situ, in real-time. Vibrational spectroscopy, as a label-free, non-invasive and non-destructive analytical technique, appears to be a promising candidate to potentially overcome many of these limitations as it can provide detailed biochemical fingerprint information for analysis of cells, tissues, and body fluids. The technique has been widely used in disease diagnosis and increasingly in stem cell technology. In this work, the efforts regarding the use of vibrational spectroscopy to identify mechanisms of stem cell differentiation at a single cell and tissue level are summarised. Both infrared absorption and Raman spectroscopic investigations are explored, and the relative merits, and future perspectives of the techniques are discussed.

## 1. Introduction

### Stem Cells and Stem Cell Technology

Stem cells are present within most, if not all, multicellular organisms and are the ultimate drivers of growth and regeneration. They can be defined as biological cells capable of self-renewal and differentiation into a variety of cell types. They have been demonstrated to play a critical role in growth and development during embryogenesis, but also in adult species for the replenishment of potentially every differentiated mature cell type [1]. 

**Figure 1:**
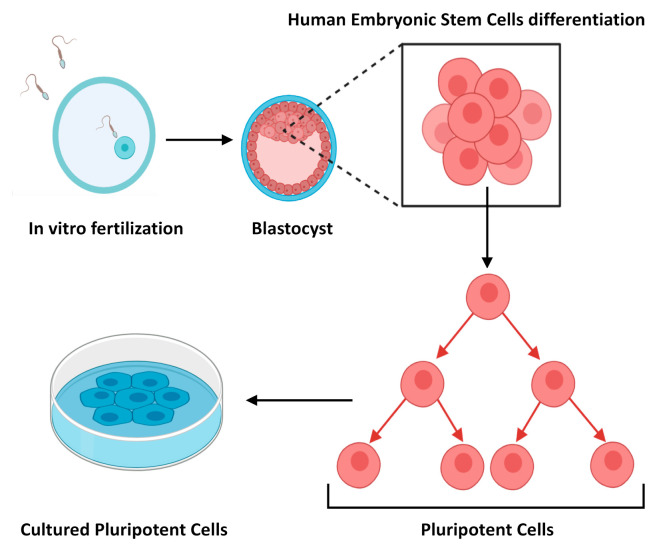
Schematic representation of how embryonic stem cells are derived.

Embryonic Stem Cells (ESCs) were first discovered and characterised in 1981 [2], and subsequently isolated from human blastocysts in 1998 [3]. Decades previously however, Pierce [4], and also Stevens and Hummel [5], demonstrated that teratocarcinomas contain cells with multi-lineage potential [6]. These findings were the first steps that led to the isolation of ESCs (Figure 1), and the identification of their extraordinary ability to proliferate indefinitely in vitro and capacity to differentiate into any somatic cell type [7]. ESCs are pluripotent cells by definition, and drive the formation of all tissues of an embryo. However, as reviewed by Lo et al., 2009 [8], the use of human ESCs for research has given rise to numerous ethical and political controversies since it involves the destruction of human embryos. Consequentially, the ethical considerations hamper their use in research. 

The identification of non-embryonic stem cells, mostly Adult Stem Cells (ASCs) [1], introduced an alternative and less controversial cell source for stem cell research and its biomedical applications. ASCs are more specialised than ESCs, and have a limited ability to differentiate to other cell types, other than the origin tissues [9]. Classified as multipotent stem cells, they are essentially committed to produce specific cell types: they have a limited, although extremely powerful ability to produce differentiated cell types, continuing to self-renew over long periods of time. Examples include those in the brain that give rise to different neural cells and glia, or haematopoietic cells, which can give rise to different blood cell types. Bone marrow also contains multipotent stem cells that give rise to all types of blood cells, but no other cell type. A number of characteristics make ASCs the most commonly used cells in regenerative medicine. The ease of the isolation process and the number of available tissues, such as umbilical cord, blood, and placenta, means that they can be readily transplanted to a site of injury without provoking immune rejection, and they naturally possess immunomodulatory properties [9].

**Figure 2:**
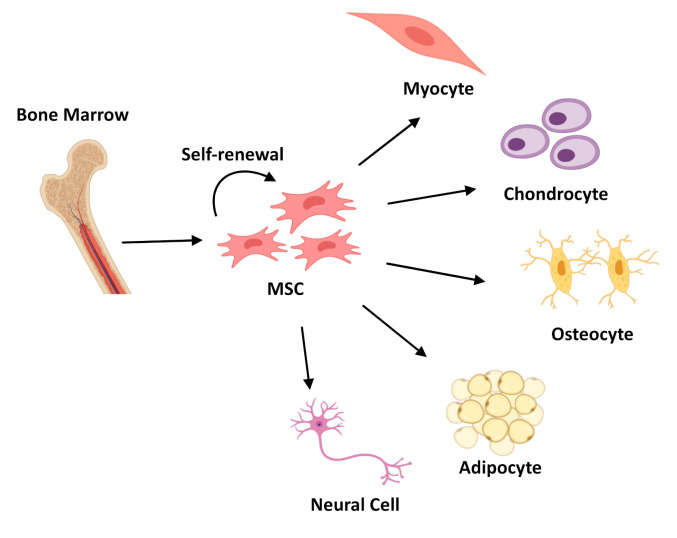
Schematic representation of Mesenchymal Stem Cells (MSCs) differentiation process into three different lineages: Myocytes, Chondrocytes, Osteocytes, Adipocytes, and Neural Cell.

Mesenchymal Stem Cells (MSCs), observed for the first time in 1968 [10], constitute approximately 30% of bone marrow [11]. They can be easily isolated from a number of tissues [9,12,13], cultured in vitro, expanded, and induced into differentiation of multiple lineages (Figure 2). Due to their remarkable capability of extensive in vitro expansion and their natural immunomodulatory properties [14], their application in modern medicine as a replacement for diseased tissues offers an excellent alternative to surgery treatments and standard medical therapies [15]. The public clinical trials database https://clinicaltrials.gov lists more than 200 clinical trials using MSCs for an extensive range of therapeutic applications including acute myocardial ischemia, stroke, liver cirrhosis, amyotrophic lateral sclerosis, and immunological diseases [16,17].

The pioneering technology of Induced Pluripotent Stem Cell (iPSC) was introduced in 2006 by Takahashi and Yamanaka [18], who showed that adult stem cells can be converted to pluripotent stem cells through the introduction of four genes [18,19] encoding specific transcription factors. iPSCs are generated by resetting the fate of somatic cells, and therefore they have the same properties as ESCs. Because of their ability to self-renew and propagate indefinitely, as well as differentiate into all cell types of the body [9], they represent a single source of cells that could be used as a replacement for a vast number of tissues. 

By the late 1990s, it became apparent that stem cells could act as a platform for the development of cell-replacement therapeutics and the fields of regenerative medicine quickly emerged. Cell-replacement therapy indeed overcomes the replacement therapy of cells en masse via organ or tissue transplant, characterised by the acute shortage of donor organs available for transplantation [20]. 

An alternative approach is ex vivo expansion of stem or progenitor cells which are then transplanted back into patients, where they undergo a process of renovating their physiological functions and the adaptive capacities in heterogeneous conditions [21,22]. 

Relevant scientific advancements, such as the identification of neural stem cells in brain tissue [23] and the first embryonic cell lines [2] set the stage for the explosion of scientific progressions in the first decade of the new millennium. By 2007, regenerative medicine and stem cells had led to significant breakthroughs, such as identification of dermal stem cells in adult skin tissues [24,25], the discovery of cancer stem cells [26], initiation of the first clinical trial of human embryonic-derived stem cells for treatment of spinal cord injuries [27,28] (Geron Corporation 2010), and many more. It is clear that recent advancements in stem cell research have provided a strong foundation of scientific knowledge setting the stage for optimistically limitless possibilities in tissues replacement therapeutics [1]. 

Establishing a fundamental comprehension of the genetic and biochemical hierarchy involved in embryonic and adult stem cell differentiation is critical in the development of new cutting-edge cell-based and non-cell-based therapeutic strategies. Determining the cell type, limiting the heterogeneity in the cell populations, and assessing and regulating the differentiation process remain some of the main challenges that must be addressed for the realisation of alternatives to surgery treatments and standard medical therapies [29]. Current methodologies for analysis of the stem cell differentiation process include immunochemistry [30], fluorescence labelling technologies [31,32,33], flow cytometry [34], reverse transcription polymerase chain reaction (rt-PCR) techniques [35,36], and RNA in situ hybridisation analysis. However, these techniques can be costly and time consuming, require significant user intervention and large volumes of cells, and are generally destructive. Monitoring the process of stem cell differentiation ideally requires continuous, in situ, characterisation of individual cells at the subcellular level, as well as population wide screening to monitor the homogeneity across a population. There is a need for novel, non-destructive, non-invasive techniques, able to provide in situ molecularly specific information at a cellular and subcellular level with a small quantity of material.

## 2. Vibrational Spectroscopy for Stem Cell Characterisation and Differentiation 

Vibrational spectroscopy has been extensively explored over the last decades as a diagnostic and prognostic tool for biomedical applications [37,38]. This technology has the capacity to provide a detailed molecularly-specific fingerprint, based on the chemical content of a specific tissue or cells [39]. FTIR has become a well-established tool in biophysics for analysis of the structure and interactions of proteins [40], lipids [41], carbohydrates [42], and nucleic acids [43]. Applications to tissue samples for (cancer) diagnostic applications were first reported in the early 1990s [44], and since this time a range of pathologies has been investigated [45,46,47]. Raman spectroscopy was first applied to biomolecules and tissues in the 1960s [48]. A variety of constituents has been measured and databases accumulated [49,50], and by the mid-1970s biomedical applications were explored [51]. Whole cell and tissue studies have been carried out on a range of pathologies [52,53,54,55,56] and in vivo studies [57,58] have demonstrated the prospective for diagnostic applications. Raman spectroscopy is able to probe molecular information from living cells revealing distinct chemical features, and biochemical processes occurring during cell culture and mitosis [59,60], proliferation [61], differentiation [62,63,64], adhesion [65], death [66,67,68,69], and invasion [70]. The subcellular resolution of vibrational spectroscopy, and in particular Raman imaging, has also been exploited to enable the detection of cellular mitochondrial distribution [60] and phagosomes [71], as well as to monitor the uptake and mechanisms of action of chemotherapeutic agents [72], and toxicity of nanoparticles [73]. It is not surprising, therefore, that the label free, non-invasive techniques have attracted increasing attention in the field of stem cell research [29] for biochemical analysis at the cellular (Section 2.1) and tissue (Section 2.2) level; the aim is to discriminate between differentiation status, detect chemical alterations before morphological changes occur, and identify the possible spectral markers in stem cells and tissues to monitor the process of stem cell differentiation in situ. 

**Figure 3:**
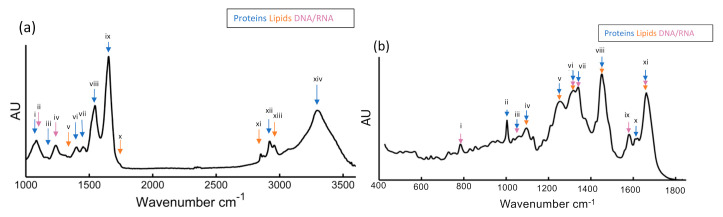
(**a**) Representative mean FTIR spectra recorded for mesenchymal stem cells (bm-MSCs) with classification of relevant peaks and their known association to proteins, nucleic acid, and lipids are described in Table 1A, (**b**) Processed mean Raman spectra for bm-MSCs and their known association to proteins, nucleic acid, and lipids are described in Table 1B [74].

Figure 3a shows an example mean FTIR spectrum of Sprague-Dawley rat mesenchymal stem cells (bm-MSC), recorded in transmission mode [74]. The more prominent bands have been labelled according to the typical biochemical origin. The main bands are labelled with accepted assignments of typical biochemical origin. The so-called “high wavenumber region”, >2500 cm^−1^, contains distinctive spectral features of N-H, C-H, and O-H of proteins, and lipids have been observed in the “high wavenumber region” (>2500 cm^−1^), while in the “fingerprint region” (<2000 cm^−1^) nucleic acids stretching vibrational modes at 1070 cm^−1^ and 1250 cm^−1^, Amide I (1650 cm^−1^) and Amide II (1520 cm^−1^), and lipids content at 1310 cm^−1^ and 1750 cm^−1^. A more detailed list of band assignments is provided in Table 1. For comparison, Figure 3b shows a mean Raman spectrum recorded from the nuclei of bm-MSCs, using a the 532 nm laser source of custom-built Raman micro-spectroscopy system described previously [75,76]. The most prominent peaks are annotated and listed in Table 1B. It should be noted that FTIR and Raman are complementary techniques; this means that although the features in the respective spectra have similar origin, vibrations of highly polar bonds (e.g., O-H) tend to be highly IR but weakly Raman active, and vice versa for vibrations of highly polarisable bonds (e.g., C=C). The high spectral dispersion of common benchtop Raman instruments means that a single detector array window captures only the fingerprint region, and commonly the high wavenumber region is not recorded. Raman micro-spectroscopy has intrinsically higher spatial resolution however, enabling subcellular analysis with the aid of multivariate statistical analysis techniques such as principal components analysis [77]. As an example, the Raman spectrum of the nucleus in Figure 3b exhibits similarly prominent signatures associated with proteins and lipids across the fingerprint region, as well as large peaks related to DNA and RNA at 785 cm^−1^.

### 2.1. Applications of IR and Raman Spectroscopy to Characteristion of Stem Cell Differentiation: Cellular Studies

#### 2.1.1. Embryonic Stem Cells 

Embryonic stem cells (ESCs) have been demonstrated to be an incomparable resource in the development of tissue engineering and regenerative medicine, due to their enormous differentiation potential in vitro. However, it is important to be able to establish and monitor differentiated/undifferentiated ratios for applications. Furthermore, alongside their immortality and self-renewal capacity, ESCs also have the capacity to differentiate in situ into tumours, such as teratomas, making their use challenging.

Amongst the earliest reports which employed vibrational spectroscopy to study ESCs were those of Notingher et al. in 2004 [64,81]. The first study investigated the biochemical changes in murine ESCs (mESCs) throughout the process of differentiation using Raman micro-spectroscopy [81]. Evolving contributions of a number of biomolecules were identified, but the greatest difference between undifferentiated mESCs differentiated was observed to be in the nucleic acid content. Spectral features of RNA and DNA, especially in the region 770–880 cm^−1^, showed a strong decrease in differentiated cells by 50%. The results suggested that the differentiated cells were more in the G1 phase of the cell cycle than S, G2, or M phases, and therefore the proliferation rate was strongly reduced in the differentiated cells, which developed a mature phenotype, compared with the undifferentiated ESCs [81]. In the second publication [64], the authors showed how mESCs induced into spontaneous differentiation could be easily be distinguished from 16–20 day old differentiated cells using Raman spectroscopy. Differentiated cells showed a different morphology and were confirmed differentiated through immunostaining assays, while the ratio between the peak areas of RNA and proteins was calculated and used as a measure of mRNA translation [64]. The nucleic acid content was observed to be strongly decreased in differentiated cells compared with their mESCs progenitors, suggesting that the concentration of RNA decreased throughout the differentiation process, following a transcription for the synthesis of specific proteins. These results provided useful insights for the identification of significant spectral markers and characterisation of ESCs and their differentiation in vitro. Some years later, Chan et al. [82] extended this work to human ESCs (hESCs), by probing with Raman their differentiation into ventricular cardiomyocytes. Consistent with the previous studies, higher nucleic acid peak intensities were observed in hESCs. The discrimination of hESCs and their progeny according to RNA levels was confirmed by Principal Components/Linear Discriminant Analysis (PCA–LDA) [82].

Ami et al. [62] performed FTIR micro-spectroscopy to monitor the spontaneous differentiation of mESCs in their early development within the first 14 days. Spectra were measured at different times of the differentiation, providing a temporal evolution of the process. Important differences were observed in the spectra after the fourth day of differentiation, such as in the amide I band (1700–1600 cm^−1^), suggesting that α-helix and β-turn secondary structures started to be expressed by the cells, and strong separations into clusters confirmed the observations. The methodology was proven to be very innovative and efficient, especially for its reproducibility at different time points. Subsequently FTIR spectroscopy was employed by Tanthanucha et al. to study the neural differentiation of mouse ESCs. [83]. The main spectral differences were observed in the nucleic acid region, with contributions also from amide and lipid related bands. Lipid content, probably as an expression of the developing phosphomembrane, interestingly was seen to decrease (CH_3_ and CH_2_ vibrations, region 2852–2959 cm^−1^), whereas glycerophospholipids levels have been showed to increase with the differentiation process [83]. Heraud et al. [84] published a study on the efficacy of FTIR as a tool to distinguish hESCs in mesodermal and ectodermal development, observing changes in the nucleic acid and protein contents after only five days of differentiation. The formation of hepatocytes has been studied with FTIR by Ami et al. [85] and with synchrotron FTIR micro-spectroscopy [85], providing detailed chemical compositions of the hepatic progenitors. Interesting variations have been identified, such as an increased amide I contribution of α-helix proteins, which has been hypothesised to reflect the emerging presence of albumin in the cells, alongside the appearance of β-sheet proteins secondary structure.

The results of the first Raman studies on mESCs [64,81,82] confirmed that the changes in RNA content in cells can be used as a marker to distinguish differentiated cells from their pluripotent progenitors. This discovery was reinforced by another study reported by Shoulze et al. [86], which non-invasively assessed the differentiation status of hESCs into heterogenic progeny with Raman spectroscopy. The study reported that it is possible to evaluate the differentiation status of the cells by determination of the intensity ratio of certain specific bands (generally contributions of nucleic acid bands, such as 757/784 cm^−1^ and 827/811 cm^−1^).

Glycogen bands were demonstrated to play an important role as a discriminant spectral marker between cardiomyocyte populations and their progenitors [87,88] and in the study published by Koronov et al. [89], Raman spectroscopy was applied to measure the absolute glycogen content of hESCs, providing a more rapid and accurate quantification compared to the commercial assay kit. Koronov et al. also reported a study of cytochemical variations between different colonies in order to understand the differentiation process [90], and a live in situ analysis of mESCs using coherent anti-Stokes Raman scattering (CARS) [91]. Moreover, CARS microscopy was employed by Downes et al. [92] to study the osteoblast mineralisation and lipid production of adipocytes as a technique capable of discriminating them over their progenitors. More recent studies have focussed on establishing the specific biomarkers identified in the differentiation process towards hepatocytes [93] and on identifying the major spectral differences between ESCs and iPSCs [94,95].

#### 2.1.2. Mesenchymal Stem Cell

The ability of MSCs to develop into various cell lineages, the ease with which they can be expanded in culture, and their innate immunomodulatory properties has led to a great deal of interest in their use as a therapeutic tool in various treatments. The highest degree of lineage plasticity has been attributed to bone marrow derived MSCs [96], which can be isolated from adult tissues and induced to differentiate into the desired lineage. However, the lack of a lineage specific and definitive marker remains one of the biggest challenges to their use.

Because of the limited supply of an allogenic source of bone and the significant emerging interest in tissue regeneration based on MSCs, some of the earliest vibrational spectroscopy studies [97,98,99,100,101] focussed on the osteogenic differentiation. In the study published in 2006 by Azrad et al. [99], Raman spectroscopy was used to study the effect of two different enhancers (QEVA and Dexamethasone) of MSCs induced to the osteogenic differentiation process. The differentiation status was demonstrated by immunochemistry and staining assays, while spectral changes during the formation and mineralisation of the bone nodules were identified in the Raman signatures. Amongst the most relevant bands, the greatest changes were observed in the range 950–960 cm^−1^, corresponding to vibrations of hydroxyapatite, suggesting the presence of calcium phosphate species (ACP amorphous cp and OCP octacalcium), which are markers for mineralisation. In another study the following year, Krafft et al. probed the osteogenesis of human MSCs with FTIR, with and without osteogenic stimulation [102]. FTIR spectroscopic signatures and differentiation markers were identified, such as that at 1100 cm^−1^, indicating the presence of calcium phosphate salts, while the undifferentiated cells (non-stimulated) showed increased levels of glycogen content (peak intensities at 1025, 1080, and 1152 cm^−1^) [102]. Gentleman et al. [97] observed the development of mineralisation nodules within 28 days of differentiation; morphological changes were illustrated by alizarin red staining and SEM imaging, while Raman micro-spectroscopic signatures of the mineralised nodules were compared with native bone. Both of them were dominated by the band at ~960 cm^−1^, corresponding to PO_4_^3−^ vibrations. Raman micro-spectroscopy has also been employed to record 28-days human MSCs derived from osteogenic differentiation by McManus et al. [100]; positive changes in collagen type II and in phosphate content were reported, and the results were validated by specific rt-PCR and staining tests. The following year, McManus et al. [101] demonstrated through Raman spectroscopy that osteoblast-like cells have similar attributes to native bone, and therefore could potentially be used as a model for human primary osteoblasts.

Adipogenic differentiation was also investigated with both FTIR [14], and surface-enhanced Raman spectroscopy (SERS) over a period of 22 days [103]. In the first study, MSCs were induced into differentiation, and pre-adipocytes were characterised at different time points. On the third and fifth days, specific peaks derived from ester bonds of triglycerides (1739 cm^−1^) appeared to be increased over the undifferentiated progenitors. Moody et al. [103] observed the adipogenesis process of human-adipose-derived stem cells over a period of 22 days, monitoring the intracellular accumulation of lipids towards the completion of the differentiation process. 

Moreover, chondrogenic differentiation of hMSCs has been monitored with synchrotron FTIR micro-spetroscopy [104] and with Raman micro-spectroscopy up to 21 days [105,106]. The major differences were observed in collagen at 1338 cm^−1^ (amide III band), PO^−^ stretching, and proteoglycan protein related bands at 1245 cm^−1^ 1065, 1079, and 1300 cm^−1^ (S–O stretching band) [104,106]. However, as MSCs develop into chondrocytes, they secrete extracellular matrix (ECM) which encompasses them, forming the dense and complex framework that constitutes the cartilaginous scaffold. Therefore chondrogenesis has been more frequently studied from the point of view of cartilage ECM formation [107], and will be discussed further in Section 2.2. FTIR has also been utilised successfully to discriminate functional hepatocytes [85] and hematopoietic stem cells [68].

FTIR has been used alongside Raman spectroscopy to discriminate de-differentiated smooth muscle cells (ddSMCs) from undifferentiated stem cells and their myogenic and osteogenic progeny, in order to better elucidate the mechanism of accumulation of SMC-like cells in the vessel and obstruct the blood flow [74]. Significant spectral differences were observed between bone marrow MSCs, their progeny, and ddSMCs by FTIR and PCA, suggesting that the cell populations are clearly distinct. The use study demonstrated the potential advantages of the complementarity of FTIR and Raman micro-spectroscopy in parallel studies, whereby the lower resolution of the FTIR imaging is used for screening of larger populations, whereas the subcellular resolution of Raman can be employed to better elucidate the underlying mechanisms of differentiation [74]. Both FTIR and Raman spectroscopy have been successfully used to distinguish between hESCs and human Mesenchymal Stem cells (hMSCs) [108]. Moreover, the complementarity of these techniques has been highlighted and consistently reviewed [29,38,109].

#### 2.1.3. Induced Pluripotent Stem Cells

iPSCs have the potential to revolutionise many aspects of stem cell science and regenerative medicine by offering multiple disease modelling and therapeutic options that were not previously possible. Recent studies have revealed a trace of epigenetic memory preserved in iPSCs, according to the somatic cell of origin [110,111], which can facilitate or hamper their differentiation fate. Their pluripotent capacity is assessed by specific phenotypic profiling, or by functional assays such as teratoma induction and global genes expression detection [112]; however methodologies able to identify iPSCs by their biophysical characteristics are still lacking.

In 2012, Tan et al. [94] published a significant study on using Raman spectroscopy for the comparison and assessment of the differences between hESCs, hiPSCs, and non-specifically differentiated hESCs over a period of 20 days. The study demonstrated that spectra of hESCs more closely resemble those of induced cells than the differentiated progeny. In fact, spectra of hiPSCs and hESCs were found to be extremely similar, and both were distinguishable from differentiated hESCs (non-specifically differentiated) in terms of relative Raman peak intensities. The main spectral differences were identified in the protein content (757 cm^−1^, 876 cm^−1^, 1003 cm^−1^, 1032 cm^−1^) of nucleic acids (783 cm^−1^) and lipids (699 cm^−1^, 717 cm^−1^) [94]. 

FTIR spectral profiles of differentiated pancreatic cells (DPCs) [113] and kidney cells (DKCs) [114] were compared with their pluripotent progenitors, and analysed at different maturation stages (11, 17, and 21 days [113], and 0, 10, 15, and 21 days [114] respectively) alongside genetic, phenotypic, and biochemical analysis by real-time quantitative PCR (RT-qPCR) and immunocytochemistry assays. PCA of FTIR spectra was used to characterise chemical and structurally mouse pluripotent stem cells (miPSCs) and their differentiation process to DKCs and DPCs. Distinct differences were observed between the cell lineages, including in the protein amide (1700–1500 cm^−1^) and carbohydrates and nucleic acid regions (1200–850 cm^−1^), a significant increase in the intensity of the bands corresponding to glycogen (1030 cm^−1^ and 1080 cm^−1^) [113,115] in DPCs, compared to miPSCs, and the C=O esteric band of lipids [114], which was seen to be more intense than in miPSCs, probably due to phosphatidylcholine synthesis throughout the metabolic kidney development. Furthermore, a recent study with Raman spectroscopy identified, at the single cell level, the most relevant differences between hiPSCs and hiPSC cell-derived neurons [116], developmental stages of the neural lineage differentiation were investigated, and the most prominent changes were found in the bands at 400 and 417 cm^−1^ (saccharides), 480 cm^−1^ (glycogen), 746 cm^−1^, 1125 cm^−1^, and 1580 cm^−1^ (cytochrome c), 720 and 780 cm^−1^ (DNA/RNA), 1003 and 1030 cm^−1^ (phenylalanine), 1295 and 1440 cm^−1^ (lipids), and 1660 cm^−1^ (proteins) [116].

**Figure 4:**
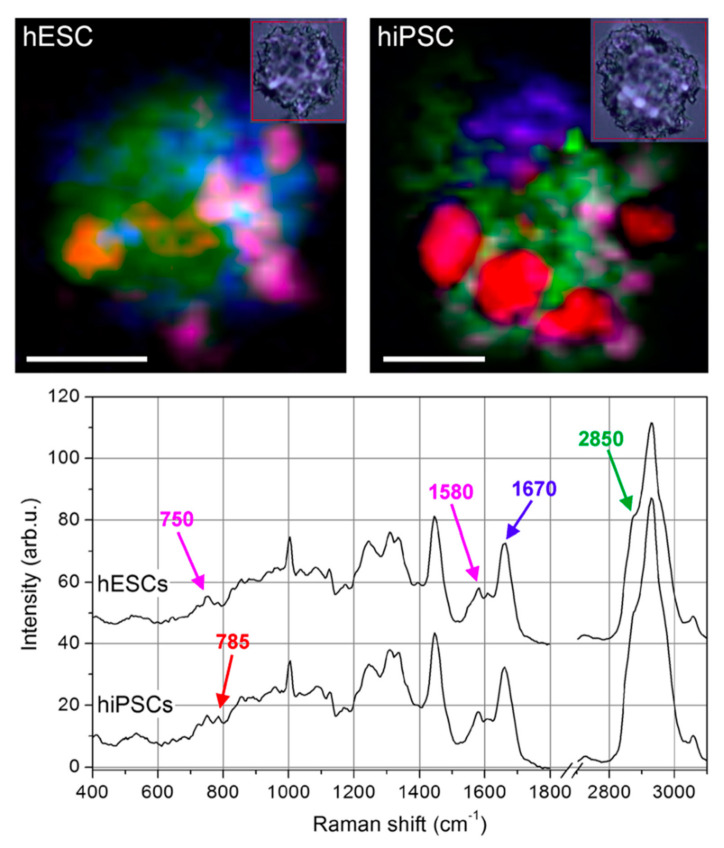
Raman imaging of typical human Embryonic Stem Cells (ESCs) and Induced Pluripotent Stem Cells (iPSCs). Colour-reconstituted Raman images of human embryonic stem cells (hESCs, upper left panel) and human induced pluripotent stem cells (hiPSCs) (upper right panel). White scale bar = 5 μm. Small insets show corresponding bright-field images recorded after Raman scanning. Raman peak at 785 cm^–1^ (DNA/RNA bases) mapped in red, 1670 cm^–1^ (proteins) in blue, 2850 cm^–1^ (lipids) in green, and a combination of 748 and 1585 cm^−1^ (cytochrome C) in magenta. hiPSCs exhibit a much higher level of DNA/RNA bases in well-defined regions of the cell. Curves in the lower panel are average spectra of hESCs (top curve) and hiPSCs (bottom curve), where the peaks used for the colour-reconstituted images are indicated with the corresponding colour (Reproduced from Parrotta et al. [95] under Creative Commons CC BY license).

Although undifferentiated ESCs and fully reprogrammed iPSCs signatures were earlier observed to be undistinguishable [94], the study by Parrotta et al. [95] of embryonic and induced pluripotent stem cells performed with Raman spectroscopy highlighted major differences between the two (Figure 4). Their main discoveries revealed differences in nucleic acid content, shown by the strong positive peaks at 785, 1098, 1334, 1371, 1484, and 1575 cm^−1^, which are enhanced in human induced pluripotent stem cells, potentially due to the different epigenetic background [95]. HiPSCs and hESCs have been previously demonstrated to express largely similar features, although metabolic differences between the two pluripotent lines have also been hypothesized [94]. These recent results demonstrated that, even though the spectral signatures of hiPSCs closely resemble those of hESCs, Raman spectroscopy was well suited for identifying the small biochemical differences that discriminate them.

### 2.2. Applications of IR and Raman Spectroscopy to Characteristion of Stem Cell Differentiation: Tissue Studies 

Vibrational spectroscopy using both FTIR and Raman spectroscopy has been used over the past few years in the study of the mechanisms underlying tissue regeneration. However, the biggest challenge with many current analytical techniques is that they often involve disrupting the sample growth, and therefore the very process under investigation (e.g., tissue sectioning, immunostaining). As a non-destructive, label-free technique, vibrational spectroscopy can determine the molecular composition, detect chemical alterations before morphological changes are evident, and discriminate between a variety of tissue types. This technology has been used to detect cellular differentiation [117] and apoptosis [118], to assess the effects of post-transplant organ regeneration [119,120], offering great promise for in vitro and in vivo diagnosis potential applications and eliminating or reducing the need for biopsies.

**Figure 5:**
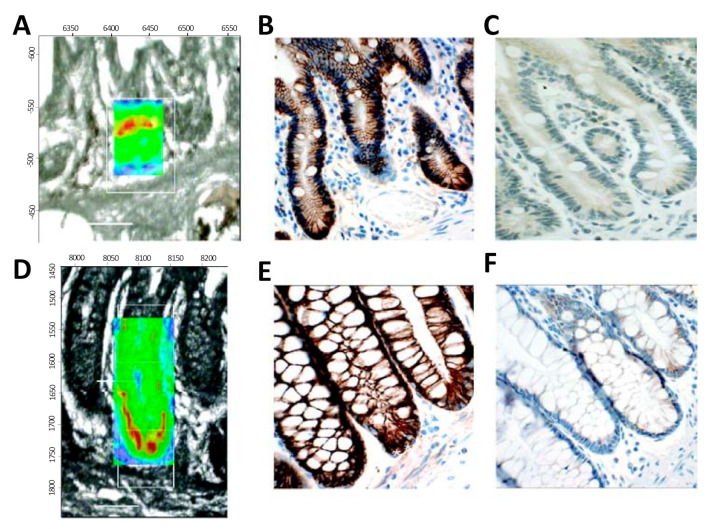
Localisation of the putative stem cell region in an IR spectral image (resolution of 8 μm × 8 μm) map of intestinal crypts obtained using synchrotron FTIR micro-spectroscopy; a comparison with proposed immunophenotypical markers. (**A**): Two-dimensional (2D) map of a small intestinal crypt, smoothed at the wavenumber 1080 cm^−1^ and superimposed on the unstained region analysed. (**B**): A representative photomicrograph of a parallel section containing small intestinal crypts stained with mouse monoclonal anti-β-catenin antibody. Note nuclear immunoreactivity in Paneth cells, identified by their pyramidal shape and “foamy” cytoplasm. (**C**): A representative photomicrograph of a parallel section containing small intestinal crypts stained with rabbit polyclonal anti-CD133 antibody. Weak immunoreactivity varied little along the length of the crypt. (**D**): 2D map of a large intestinal crypt, smoothed at the wavenumber 1080 cm^−1^ and superimposed on the unstained region analysed. (**E**): A representative photomicrograph of a parallel section containing large intestinal crypts stained with mouse monoclonal anti-β-catenin antibody. In a single crypt two nuclei (*) showed weak nuclear immunoreactivity. (**F**): A representative photomicrograph of a parallel section containing large intestinal crypts stained with rabbit polyclonal anti-CD133 antibody. Weak cytoplasmic immunoreactivity was observed throughout the length of the crypt. Spectral intensity is represented as thermal color changes (blue < green < yellow < red). The synchrotron FTIR micro-spectroscopy experiments were carried out at the European Synchrotron Radiation Facility. (Reproduced with permission [122]).

Stem cells of the intestinal crypt have been widely investigated, aiming to identify reliable markers for accurate discrimination of stem cell location from the transient-amplifying (TA) cells within the crypts, and differentiated cells [121]. In 2008 [122], FTIR spectroscopic imaging was used to identify the locations and spectral signatures of stem cell regions of human intestinal crypts (Figure 5). Spectral mapping was used, and significative differences within the three tissue locations were identified, especially for the vibrations of DNA (1225 cm^−1^, 1080 cm^−1^). Small and large intestinal crypts showed the most intense PO^−^ signals, which were identified as the most robust IR spectral marker for stem cell identification. Bentley et al. [123] and Kelly et al. [124] successfully investigated the human corneal epithelium with synchrotron-based FTIR spectroscopy, revealing the major biomarkers responsible for the distinction between TA cells, terminally-differentiated cells (TD), stem cells, and corneal squamous cell carcinoma [124]. Differences were identified between TA, TD, and stem cells due to protein associated peaks and RNA expression [123], and carbohydrate and glycogen regions [124]. Moreover a spectral profile of squamous cell carcinoma cells was defined, defining their highly proliferative nature and capability of giving rise to a TA cell-like class [124].

In recent years, there has been a growing interest in the use of vibrational spectroscopy to characterise the extracellular matrix (ECM). A complex network of biomolecules, such as glycosaminoglycans (GAGs), proteoglycans, and collagen are deposited in the ECM of tissues, and the possibility to specifically assess these elements can be applied across a broad range of applications, including tissue engineering [125]. Raman spectroscopy represents a particularly powerful approach for bone ECM characterisation, as reported by Draper et al. [126] and Mandair et al. [127], who used this technology extensively to analyse how changes in bone composition and structure influence tissue level bone mechanical properties. Bone ECM has a highly crystalline mineral structure, which exhibits very strong Raman scattering signal, especially the band ∼960 cm^−1^ (ν1 PO^3−^), characteristic of the apatite crystal content. Furthermore, FTIR spectroscopy has been used to establish a new protocol for high quality bone formation and characterization, with the potential to be applied to bone tissue engineering [128].

The cartilaginous ECM is extremely rich in collagen type I and IX, and XI fibers [129], interspersed with GAG macromolecules, and it is produced and maintained by the chondrocytes. The loss of ECM homeostasis and the degradation of cartilage lead to severe diseases, such as osteoarthritis (OA) [130], making the detection of the involved factors a matter of great importance. Lim et al. [131] used Raman micro-spectroscopy to study the biomolecular changes associated with cartilage damage prior to visible histological changes, observing a decrease in the GAGs collagen (Amide III 1264–1274 cm^−1^) content, in early cartilage damage. Pudlas et al. [106] identified the chondrocytes differentiation state by the study of proteoglycans and GAGs in different zones of cartilaginous ECM, both porcine and human, recognising well-defined Raman spectral biomarkers. Major bands at 1065 (SO^−3^), 1079 (C–C stretch vibrations), and 1300 cm^−1^ (lipids) were identified and classified of high interest for the differentiation status assessment [106].

A recent study using Raman spectroscopy analysed the higher wavenumber region of the spectrum between 2700 and 3800 cm^−1^, in which the peaks were seen to be correlated with increases in the permeability and variations in the aggregate modulus of articular cartilage [132]. The study indicates the water content in human articular cartilage can be (inversely) correlated with the mechanical properties of cartilage and could potentially be used as an in vivo probe of diseased tissues (OA associated). 

## 3. Future Perspectives on the Use of Vibrational Spectroscopy for Stem Cell Technology; Data Analysis and Machine Learning, 3D Cultures and Nanotechnology

Research on regenerative medicine and development of novel therapeutic targets has immensely accelerated following the introduction of stem cell technologies. Following the identification of different types of stem cells, their potential to differentiate into different lineages has led to escalated research efforts, and stem cell technology has attracted considerable attention due to its potential to extend recovery capabilities of the human body following many diseases and injuries which are not currently treatable by current medical procedures. Despite their enormous potential in clinical research, these new technologies still hold many unknowns, and applications remain limited by a lack of fundamental understanding of their differentiation mechanisms and ability to fully regulate the triggering of their differentiation. The importance of the use of multi-disciplinary approaches by employing new emerging technologies to understand their mechanism of action is therefore beyond question. In this review, we aimed to introduce the different types of stem cells and their potential uses for clinical applications, as well as collective efforts to date to employ vibrational spectroscopic techniques, both at the single cell and tissue level, to understand and control their mechanism of differentiation. Undoubtedly, there is still room for improvement and research efforts will continue in an attempt to harness the full benefits of stem cell technology. This section will focus on the future perspectives and possibility of extending current applications and incorporation of other technologies to improve our understanding of the differentiation process.

Identification of stem cells in complex biological environments, based on their biochemical characteristics and investigation of spectral markers of the differentiation process have been amongst the main targets of stem cells research. In the context of basic research and proof-of-concept studies, vibrational spectroscopy has provided information regarding the biochemical composition of stem cells and introduced spectral markers to monitor differentiation, in a label free and non-destructive way. However, conflicting or inconsistent results from the studies regarding identified biomarkers and commonality of markers at different differentiation stages has made the applications limited. Therefore, more systematic approaches, a cross-check of differentiation markers during differentiation into various lineages and time dependant investigation of spectral marker evolutions will be critical to move forward. Notably, while spectral signatures of biomolecules such as RNA, DNA, proteins carbohydrates, and lipids have been identified at subcellular, cellular, ECM, and tissue levels, a holistic spectroscopic picture of the biochemical evolution from stem cell triggering, through the differentiation process, to tissue formation, is still lacking. Ultimately, protocols for continuous in situ monitoring of live stem cell differentiation, maturation, and tissue formation are required, but in the fields of both IR and Raman spectroscopy, novel technologies continue to emerge. Traditionally, it is considered that the relatively strong absorption of water in the mid infrared region favours the use of Raman, rather than IR, micro-spectroscopy for in vitro monitoring [39]. Live cell imaging has been performed in vitro using higher power sources such as synchrotrons [133], and emerging technologies based on quantum cascade lasers have brought such higher powers to the benchtop for more routine infrared analysis of cells and tissues [134]. Water is a relatively weak scatterer, and live cell imaging is relatively routine, at least over limited time periods [135]. While standardisation of interlaboratory protocols for Raman spectroscopic characterisation of biological materials is still under development [136], instrumentally, the principle challenge is that of the sample containment, in an appropriate viable environment. Microscope stage incubation units for live cell measurements are increasingly available [88,137,138,139], and Raman spectroscopic systems themselves continue to evolve. Fibre based systems are increasingly being evolved for high spectral quality in situ, and even in vivo biomedical applications [38], and thus the prospect that stem cells can be identified, and their evolution monitored in an in vivo environment, is real.

Beyond basic research, the prospective applications of Raman spectroscopy in the development of cell therapies has recently been reviewed by Rangan et al. [140]. It is noted that the broad impact benefit is potentially at the stages of monitoring the stages of cell harvesting, cell expansion, and cell generation. Numerous studies on stem cells [15,86,89,91], and analysis of the cells of the immune system [141] have validated the applicability of Raman spectroscopy to performing highly specific cell characterisation. High throughput applications have also been demonstrated [142], and therefore it can be anticipated that the technique can play a role in analysing and validating the quality of therapeutic cells, and so in monitoring the purification, enrichment, and isolation of cell populations. Raman spectroscopy is already implemented in a number of areas as an online tool for process analytical technology (PAT) [143], and thus can also be employed for routine, automated monitoring of microbial or xenobiotic contamination in scaled up manufacturing processes. 

Realisation of clinical applications of vibration spectroscopy have, to date, been relatively slow [38]. However, the areas of analysis of blood for disease diagnostics, as well therapeutic monitoring, have been particularly active of late [144,145,146], as has that of in vivo probes for both diagnostic applications and intraoperative determination of resection margins [147,148,149]. It can be projected therefore that vibrational spectroscopy can add value to the cell therapy pipeline, to monitor therapeutic efficacies and immunoresponses.

Moreover, introduction of advanced data analysis techniques and machine learning strategies to extract the information from vibrational spectroscopy data sets can bring new insights into stem cell technology. Pre-processing and data analysis have been accepted to be undeniable components of vibrational spectroscopic techniques in order to improve spectral quality by reducing background and measurement artefacts, as well as to extract biochemical and hierarchical information from data sets. The complexity of the measured environment combined with the dimensionality of data sets led researchers to apply non-supervised and supervised multivariate analysis techniques, such as principal component analysis, partial least squares regression and partial least-squares-discriminant analysis. 

It should also be noted that, to date, research on stem cells utilising vibrational spectroscopy has mainly been performed on two-dimensional (2D) cultures. Although 2D in vitro culture models have been widely used due to their convenience and high cellular viability and provided valuable information about the variety of cellular processes [150], they lack the complexity of the in vivo biological environment in which cells grow [151]. In vivo, cells are surrounded by an extracellular matrix with acts as a support, provides mechanical properties and facilitates communication between cells [152]. This surrounding organisation of the in vivo environment strongly influences cell behaviours such as proliferation, metabolism, survival, resistance to therapeutics, and differentiation in the case of stem cells [153,154]. Therefore, the use of 3D substrates and/or co-cultures in spectroscopic studies of stem cells should be explored more extensively.

Additional efforts to understand their mechanism of differentiation, identification of biomarkers, and spectral markers of differentiation will open a new door to control their differentiation processes and ultimately will bring new insights to their clinical use. In this respect, merging stem cell technologies with other emerging technologies, such as nanotechnology, can provide new strategies to control and direct their differentiation. The ideas of using nanomaterials for biomedical applications and to understand cellular dynamics have gained immense interest [155]. Their unique physicochemical and optical properties, as well as ease of interaction with biological materials and living systems, has precipitated the idea of using them as drug carrying agents and targeting agents. Most nanoparticles have been shown to be taken up readily by cells and trafficked through subcellular compartments. In addition to their transport inside the cells, these nanomaterials have been shown to carry messages and activate and/or inhibit biological processes inside the cell. The effect of nanoparticles on the suppression and/or enhancement of the differentiation has been shown by various groups (for a detailed review on the topic, please see Dayem et al., 2016) [156]. Gold nanoparticles have been demonstrated to enhance the differentiation of stem cells into osteogenic and neural lineages, whereas they suppress adipogenic differentiation [157,158]. Moreover, in individual studies, the enhancing effects of silver nanoparticles and dexamethasone-loaded carboxymethyl chitosan/polyamidoamine dendrimers on differentiation have been shown, albeit based on different mechanisms of action [159,160]. Based on the research to date, it is not unreasonable to predict that using nanomaterials during the differentiation process will show an impact on the overall process and could change the commitment of the stem cell. In the future, more systematic investigation of nanoparticle-stem cell interactions can provide more insight to their clinical applications.

In all aspects of stem cell research, both Raman and FTIR micro-spectroscopies have demonstrated their potential to contribute additional insight into the process of stem cell triggering, the evolution of cellular phenotypes during differentiation, and the process of tissue generation. The non-invasive, label free nature of the techniques means that either can be employed to monitor cell and/or tissue growth in situ. The complementarity of the two techniques has been demonstrated to integrate the capacity to perform studies from subcellular to large population screening. Beyond fundamental research, potential clinical applications have already been explored, with the promise of translation to in vivo monitoring of tissue regeneration and stem cell therapeutics.

## Figures and Tables

**Table 1:** Peak assignments for (**A**) FTIR spectra data (**B**) Raman spectra data [15,39,63,78,79,80].

A	Wavenumber	FTIR Peak Assignments	Association
**(i)**	1036	C-C skeletal stretching	Proteins
**(ii)**	1072	PO_2_ symmetric stretching	DNA/RNA
**(iii)**	1152	C-C and C-O stretching	Proteins
**(iv)**	1220–1280	PO_2_ asymmetric stretching Amide III	DNA/RNA
**(v)**	1312	CH_2_ stretching	Phospholipids
**(vi)**	1400	CH_3_ symmetric stretching	Proteins
**(vii)**	1456	CH_3_ asymmetric stretching	Proteins
**(viii)**	1546	Amide II	Proteins
**(ix)**	1620–1700	Amide I	Proteins
**(x)**	1742	Ester, C=O stretching	Lipids
**(xi)**	2854	CH_2_ symmetric stretching	Lipids
**(xii)**	2926	CH_2_ asymmetric stretching	Lipids
**B**	**Wavenumber**	**Raman Peaks Assignment**	**Association**
**(i)**	785–788	Stretching of DNA/RNA related	Nucleic Acid
**(ii)**	1004	Phenylalanine	Protein
**(iii)**	1090	Stretching of DNA related bonds Stretching of C-N backbone	Nucleic Acid Protein
**(iv)**	1127	Stretching of C-N backbone Stretching of C-C	Protein Lipid
**(v)**	1262	DNA/RNA breathing modes Amide III	Nucleic Acid Lipid
**(vi)**	1319	CH_2_, CH_3_ twisting DNA/RNA breathing modes CH deformation vibration	Lipid Nucleic Acid Protein
**(vii)**	1341	DNA/RNA breathing modes CH deformation vibration	Nucleic Acid Protein
**(viii)**	1451	CH_2_ deformation vibration	Protein/Lipid
**(ix)**	1585	DNA/RNA breathing modes	Nucleic Acid
**(x)**	1619	Tyrosine; tryptophan	Protein
**(xi)**	1662	DNA/RNA breathing modes Amide I Fatty acids	Nucleic Acid Protein Lipid
